# Methamphetamine use and suicide risk: a comprehensive case–control study

**DOI:** 10.3389/fpubh.2025.1593935

**Published:** 2025-07-24

**Authors:** Ahmed Hamed Aljadani, Mohamed Abouzed, Halima Adam, Benayan Bani Alrasheedy, Muath Alarfaj, Salwa Rashed Alshammari, Mohammed Saad Almuqahwi

**Affiliations:** ^1^College of Medicine, University of Ha’il, Hail, Saudi Arabia; ^2^Department of Psychiatry, College of Medicine, Al-Azhar University, Cairo, Egypt; ^3^Psychology Department, Education College, University of Ha’il, Hail, Saudi Arabia; ^4^Eradah Complex for Mental Health, Hail, Saudi Arabia; ^5^Eradah Complex for Mental Health, Dammam, Saudi Arabia

**Keywords:** methamphetamine, addiction, suicide, suicidality, risk, predictor, stimulant

## Abstract

**Introduction:**

Potent stimulants, such as methamphetamine, exert powerful psychological and physiological effects that could affect suicide risks. Therefore, the aim of this study was to investigate whether methamphetamine use is independently associated with suicidality after adjusting for potential confounding variables.

**Methods:**

This case–control study investigated the independent role of methamphetamine use in suicidal ideation, suicide planning, and suicide attempts in 800 patients who were substance users receiving treatment from mental health hospitals in Saudi Arabia. Methamphetamine users and non-users comprised the case and control groups, respectively. Urine analyses were performed to verify the participants’ self-reported drug use, and the Columbia-Suicide Severity Rating Scale, Addiction Severity Index, and Timeline Follow-Back approach were used in the data-gathering process. Multivariate analyses, adjusted for confounding factors, were conducted to assess the relationship between methamphetamine use and suicidality. Kaplan–Meier survival analysis was used to determine the survival probabilities of methamphetamine users and non-users.

**Results:**

The proportion of methamphetamine users with a history of suicidality was significantly higher than that of non-users (30% vs. 20%, respectively; *p* < 0.001), and a higher proportion of users attempted suicide (18% vs. 7%). The dose–response relationship revealed adjusted odds ratios increasing from 1.8 (95% confidence interval (CI): 1.3–2.5) in light users (1–2 times monthly) to 3.2 (95% CI: 2.3–4.4) in heavy users (every day), demonstrating that daily methamphetamine use significantly increased suicide risk. Compared with that of non-users, methamphetamine users had a significantly poorer survival rate (hazard ratio = 2.4, *p* < 0.001). Hanging was the most common method of suicide, followed by self-poisoning.

**Conclusion:**

Methamphetamine use was identified as a strong independent predictor of suicidality. These findings underscore the critical need for comprehensive evaluations in mental health care, as well as tailored interventions and long-term monitoring of users to reduce the suicide risk in this vulnerable population.

## 1 Introduction

Methamphetamine, a highly addictive psychostimulant, is among the most commonly used illicit substances worldwide and is responsible for a considerable proportion of all drug-related mortality and morbidity (1). Geographically, methamphetamine use is most common in North America, South America, and Asia, with increasing expansion to the Middle East and North Africa (MENA) region, reflecting global trends in stimulant consumption (2). Smoking is the preferred route of administration, followed by intravenous injection, with both methods contributing to a rapid onset of action, strong addictive potential, and compulsive use. Chronic methamphetamine use is associated with serious adverse effects on physical, mental, and social health, and the related neurotoxicity aggravates psychiatric symptoms and cognitive deficits while promoting behavioral dysregulation (3, 4). Methamphetamine, popularly known as “Shabu,” has been identified as an increasing concern in Saudi Arabia (5).

Although methamphetamine use is known to elevate the risk of various physical and psychiatric outcomes, its association with suicidality warrants further investigation. There is evidence to suggest that both short- and long-term use might affect mood regulation and impulse control, both of which are significant risk factors for suicide (6, 7); however, the methodological constraints of past observational studies have prevented the research community from achieving a comprehensive understanding of the directionality and independence of this association.

Suicide has been estimated to lead to more than 700,000 deaths annually, making it a leading cause of preventable death worldwide. In Saudi Arabia, the lifetime prevalence rates for suicidal ideation, planning, and attempts were 4.90, 1.78, and 1.46%, respectively. The corresponding 12-month prevalence rates were 1.82, 0.89, and 0.63% (8). Substance use disorders (SUDs) are known to play an important role in influencing suicide risk and significantly contribute to global mortality. For example, individuals with SUDs have a remarkably higher risk of suicidal ideation than those without SUDs, and they are nearly three to four times more likely to have suicidal thoughts and engage in suicidal behaviors; among adolescents, this association is even greater, with those affected facing a four-to-five-fold higher risk (9). Methamphetamine use has surged, with a > 500% increase in related deaths in Jeddah, KSA (2016–2018), where 21% of cases involved suicide, and polysubstance abuse (e.g., Captagon, heroin) is common (10). However, there remains a paucity of detailed research examining the burden and specific pathways linking methamphetamine use to suicidality in this cultural and epidemiological context. The association between methamphetamine use and suicidality may operate through both direct and indirect pathways. Direct neurobiological effects of methamphetamine on dopaminergic and serotonergic systems can impair mood regulation, impulse control, and cognitive function, increasing vulnerability to suicide (11). Indirectly, methamphetamine use is often accompanied by psychiatric comorbidities such as depression, psychosis, and heightened impulsivity, which are themselves well-established risk factors for suicide (12). Despite this, the extent to which the relationship between methamphetamine use and suicidality is independent or mediated through these intermediate factors remains inadequately understood, particularly in the MENA context. Given these gaps, this study aims to investigate the independent association between methamphetamine use and suicidality among patients receiving treatment in mental health hospitals in Saudi Arabia, while adjusting for potential confounders including psychiatric comorbidities and demographic factors. Understanding this relationship is critical for developing culturally appropriate prevention and intervention strategies tailored to this vulnerable population.

## 2 Methods

### 2.1 Study design

This case–control study evaluated the association between methamphetamine use and suicidality among patients who received treatment at Eradah Mental Health Hospitals in Saudi Arabia, with the main objective being to determine whether methamphetamine use is an independent contributor to suicidal thoughts, suicide planning, and suicide attempts, while accounting for other substance use patterns, psychiatric comorbidities, and demographic factors.

### 2.2 Study setting and population

This study was conducted at Eradah Mental Health Hospitals, a specialist mental health institutes (4 hospitals participated) in Saudi Arabia, encompassing both inpatient and outpatient units. Recruitment occurred between January 1, 2024, and December 31, 2024. We employed a consecutive sampling approach, whereby all patients presenting to the relevant units and meeting initial eligibility criteria during this period were systematically screened and invited to participate. This strategy aimed to reduce selection bias and enhance representativeness. The screening workflow involved initial electronic medical record review, followed by clinical eligibility assessment and informed consent discussion.

### 2.3 Eligibility criteria

Participants aged 18 years or older with a current diagnosis of Substance Use Disorder (SUD) based on DSM-5 criteria were included. Permitted DSM-5 diagnoses comprised methamphetamine use disorder, cannabis use disorder, pregabalin use disorder, alcohol use disorder, and other amphetamine use disorders. Patients with severe cognitive impairment were excluded; this was operationalized as a Mini-Mental State Examination (MMSE) score below 24 or inability to provide informed consent. Psychiatric comorbidities were clinically verified by board-certified psychiatrists via structured clinical interviews (SCID-5) and corroborated through medical record review.

### 2.4 Recruitment process

Recruitment involved systematic review of electronic health records followed by direct approach to eligible patients by trained research personnel. Both inpatient and outpatient settings were included to ensure sample diversity.

### 2.5 Data collection

Data collection employed a multimodal approach: Structured questionnaires collected demographic, clinical, and substance use information. The Arabic-validated Columbia-Suicide Severity Rating Scale (C-SSRS) assessed suicidality. The Addiction Severity Index (ASI) and Timeline Follow-Back (TLFB) approach measured substance use severity and frequency. Urine samples for methamphetamine detection were collected on admission n or during outpatients visiting. Initial screening used immunoassays with a cutoff value of 500 ng/mL. Positive results were confirmed by gas chromatography–mass spectrometry (GC–MS) to minimize false positives. Interviewers underwent a two-week training program focused on standardized administration of C-SSRS, ASI, and TLFB. Inter-rater reliability was assessed in 30 randomly selected interviews, yielding intraclass correlation coefficients (ICCs) of 0.85 (C-SSRS), 0.83 (ASI), and 0.80 (TLFB).

### 2.6 Sample size calculation

The sample size of 800 participants was determined using G*Power software based on the following parameters: *α* = 0.05 (two-tailed); power (1-*β*) = 0.80; expected effect size (OR) = 2.0; and anticipated prevalence of suicidal ideation in the non-exposed group = 15%.

### 2.7 Statistical methods

Categorical variables are expressed as frequencies and percentages, continuous variables as means ± SDs. Chi-square and independent *t*-tests assessed group differences. Multiple logistic regression models examined associations between methamphetamine use and suicidality, adjusting for covariates including age, sex, other substance use categories, and psychiatric comorbidities. Covariate retention criteria included *p* < 0.1 in univariate analysis or clinical relevance. Multicollinearity was assessed through Variance Inflation Factors (VIF), all below 3, indicating no significant collinearity. Model fit was evaluated via Hosmer–Lemeshow goodness-of-fit test (*p* > 0.05) and area under the ROC curve (AUC). Missing data ranged from 0 to 5%. Due to low missingness, listwise deletion was used primarily. Multiple imputation (five datasets) was performed as sensitivity analysis, with consistent results.

Kaplan–Meier survival analysis compared survival probabilities between groups, with log-rank tests assessing differences. Proportional hazards assumptions were tested using Schoenfeld residuals, which showed no violations. No time-varying covariates were included.

All analyses were conducted using SPSS v19. Statistical significance was set at *p* < 0.05. Benjamini–Hochberg correction was applied to control for multiple testing.

## 3 Results

The study included a total of 800 participants, equally divided into 400 methamphetamine users and 400 non-users. Methamphetamine users were slightly younger, with a mean age of 32.4 ± 7.9 years compared to 33.8 ± 8.8 years among non-users (*p* = 0.021). The overall sample was predominantly male, constituting 77% of participants, with methamphetamine users having a higher proportion of males (82%) than non-users (72%) (*p* = 0.003). A history of psychosis was significantly more common among methamphetamine users (16.8%) than non-users (7.8%) (*p* < 0.001). Additionally, suicidality was more prevalent in methamphetamine users (30%) compared to non-users (20%) (*p* < 0.001) (see Table 1).

**Table 1:** Demographic and clinical characteristics of participants (*N* = 800).

Characteristic	Total (*N* = 800)	Methamphetamine users (*N* = 400)	Non-users (*N* = 400)	*p*-value
Age (mean ± SD)	33.1 ± 8.4	32.4 ± 7.9	33.8 ± 8.8	0.021
Sex (Male), n (%)	616 (77.0%)	328 (82.0%)	288 (72.0%)	0.003
History of Psychosis, *n* (%)	98 (12.3%)	67 (16.8%)	31 (7.8%)	<0.001
History of Suicidality, *n* (%)	200 (25.0%)	120 (30.0%)	80 (20.0%)	<0.001

When evaluating types of suicidality, methamphetamine users showed a distinctive profile: suicidal ideation was less frequent in users (60%) versus non-users (82%) (adjusted OR = 0.3, 95% CI: 0.2–0.5, *p* < 0.001), but suicide planning (22% vs. 11%) and suicide attempts (18% vs. 7%) were significantly higher among users (adjusted OR = 2.3, 95% CI: 1.6–3.3 and adjusted OR = 2.9, 95% CI: 1.8–4.6, respectively) (Table 2).

**Table 2:** Prevalence of suicidality among methamphetamine users and non-users (adjusted for confounders).

Suicidality type	Methamphetamine users (*n* = 120)	Non-users (*n* = 80)	Adjusted OR (95% CI)	*p*-value
Suicidal ideation	72 (60.0%)	66 (82.0%)	0.3 (0.2–0.5)	<0.001
Suicide planning	26 (22.0%)	9 (11.0%)	2.3 (1.6–3.3)	<0.001
Suicide attempts	22 (18.0%)	6 (7.0%)	2.9 (1.8–4.6)	<0.001

Adjusted for age, sex, other substance use, and psychiatric comorbidities. OR, odds ratio; CI, confidence interval.

**Figure 1:**
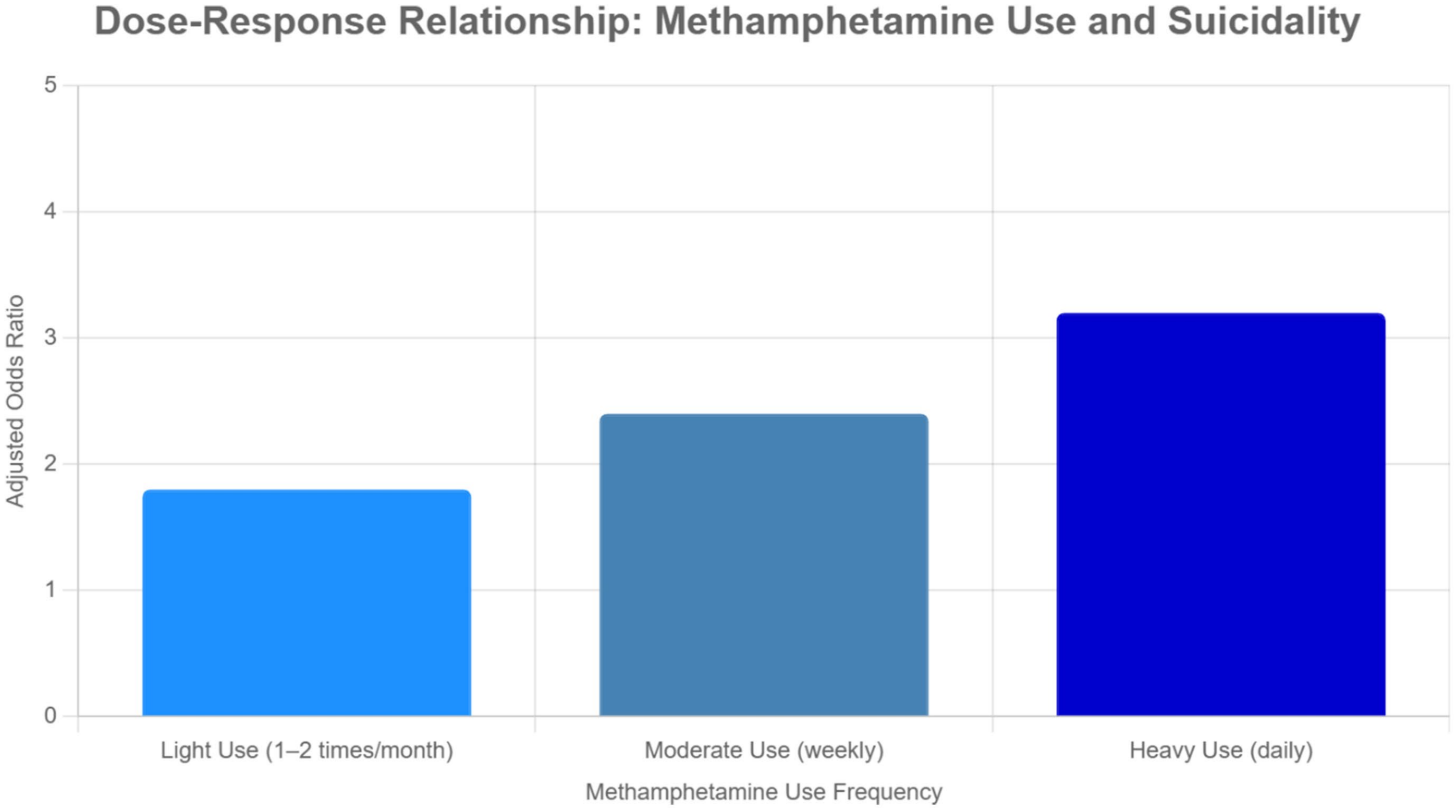
Dose–response relationship between methamphetamine use and suicidality risk. Adjusted odds ratios (ORs) with 95% confidence intervals for suicidality (composite outcome: suicidal ideation, planning, or attempts) by methamphetamine use frequency. ORs are adjusted for age, sex, other substance use, and psychiatric comorbidities. The Cochran-Armitage trend test indicates a significant dose–response relationship (*p* < 0.001). Error bars represent 95% CIs.

A significant dose–response relationship was observed between methamphetamine use frequency and suicidality risk (Cochran-Armitage test, *p* < 0.001). Adjusted odds ratios rose progressively from 1.8 (95% CI: 1.3–2.5) for light users (1–2 times/month) to 3.2 (95% CI: 2.3–4.4) for heavy daily users, as illustrated in Figure 1 and detailed in Table 3.

**Table 3:** Dose–response relationship between methamphetamine use frequency and suicidality risk (*N* = 120).

Methamphetamine use frequency	Number	Suicidal thoughts (%)	Suicide planning (%)	Suicide attempts (%)	Adjusted OR (95% CI)*
Light use (1–2 times/month)	40	30.4%	22.0%	12.8%	1.8 (1.3–2.5)
Moderate use (weekly)	50	38.7%	24.5%	16.5%	2.4 (1.7–3.3)
Heavy use (daily)	30	53.1%	30.0%	24.7%	3.2 (2.3–4.4)

*p for trend < 0.001. Adjusted for age, sex, other substance use, and psychiatric comorbidities. OR, odds ratio; CI, confidence interval.

Regarding suicide methods, hanging was the most common (71.4%), followed by self-poisoning (17.9%) and other violent methods (10.7%). There were no significant sex differences in the method distribution (Table 4).

**Table 4:** Methods of suicide attempts by sex (*N* = 28).

Method	Males (*n* = 24)	Females (*n* = 4)	Total (*n* = 28)	*p*-value
Hanging	17 (70.8%)	3 (66.7%)	20 (71.4%)	0.821
Self-Poisoning	4 (16.7%)	1 (33.3%)	5 (17.9%)	0.918
Other Violent Methods*	3 (12.5%)	0 (0.0%)	3 (10.7%)	0.684

*Other violent methods include firearms, jumping from heights, cutting or stabbing, drowning, vehicular impact, burning/self-immolation, electrocution, and ingestion of corrosive substances.

The concurrent substance use analyses revealed that methamphetamine (60%), amphetamines (40%), and sedative-hypnotic agents (30%) were prevalent among patients who exhibited suicidality. Methamphetamine (OR = 3.5, 95% CI: 2.3–5.3), opioids (OR = 4.9, 95% CI: 3.1–7.7), and antidepressants (OR = 4.2, 95% CI 2.8–6.3) were strongly associated with the selection of self-poisoning as a method of suicide (Table 5).

**Table 5:** Concurrent medication and substance use among patients with suicidality (*N* = 200) and association with self-poisoning.

Substance/medication	Total sample (*n*, %)	Self-poisoning cases OR (95% CI)	*p*-value
Methamphetamine	120 (60.0%)	3.5 (2.3–5.3)	<0.001
Alcohol	40 (20.0%)	1.8 (1.2–2.7)	0.004
Cocaine	30 (15.0%)	1.5 (1.0–2.3)	0.045
Heroin	25 (12.5%)	2.2 (1.4–3.5)	0.001
Amphetamines	80 (40.0%)	2.0 (1.4–2.9)	<0.001
Sedative-Hypnotic Agents	60 (30.0%)	2.8 (1.9–4.1)	<0.001
Antidepressants	50 (25.0%)	4.2 (2.8–6.3)	<0.001
Antipsychotics	25 (12.5%)	2.1 (1.4–3.2)	0.001
Cannabis	30 (15.0%)	1.5 (1.0–2.2)	0.045
Opioids	40 (20.0%)	4.9 (3.1–7.7)	<0.001

Logistic regression models confirmed methamphetamine use as a significant predictor: it decreased the odds of suicidal ideation (adjusted OR = 0.4, 95% CI: 0.2–0.7, *p* < 0.001) but increased the odds of suicide planning (adjusted OR = 2.3, 95% CI: 1.6–3.3, *p* < 0.001) and suicide attempts (adjusted OR = 2.9, 95% CI: 1.8–4.6, *p* < 0.001) (Table 6). Age and sex were not significant predictors after adjustment.

**Table 6:** Logistic regression results for suicidality outcomes.

Predictor	Outcome	Unadjusted *β* (SE)	Unadjusted OR (95% CI)	*p*-value	Adjusted *β* (SE)	Adjusted OR (95% CI)	*p*-value
Methamphetamine use	Suicidal ideation	−0.92 (0.25)	0.4 (0.2–0.7)	<0.001	−0.92 (0.26)	0.4 (0.2–0.7)	<0.001
Methamphetamine use	Suicide planning	0.83 (0.22)	2.3 (1.6–3.3)	<0.001	0.83 (0.22)	2.3 (1.6–3.3)	<0.001
Methamphetamine use	Suicide attempts	1.06 (0.24)	2.9 (1.8–4.6)	<0.001	1.06 (0.24)	2.9 (1.8–4.6)	<0.001
Age	Suicidal ideation	0.02 (0.01)	1.02 (1.00–1.04)	0.08	0.01 (0.01)	1.01 (0.99–1.03)	0.12
Sex (Male)	Suicide attempts	0.26 (0.20)	1.3 (0.9–1.9)	0.15	0.22 (0.21)	1.2 (0.8–1.8)	0.18

Stratified analyses by sex and psychosis history showed that methamphetamine users with psychosis had the highest risk of suicide attempts (OR = 4.1, 95% CI: 2.5–6.7, *p* < 0.001), followed by males (OR = 3.1, 95% CI: 1.9–5.0, *p* < 0.001) and females (OR = 2.7, 95% CI: 1.5–4.8, *p* = 0.001), with no significant interaction effects (Table 7).

**Table 7:** Stratified analyses by sex and psychosis history.

Subgroup	Methamphetamine Users (OR, 95% CI)	*p*-value	Interaction *p*-value
Males	3.1 (1.9–5.0)	<0.001	0.42
Females	2.7 (1.5–4.8)	0.001	
Psychosis history	4.1 (2.5–6.7)	<0.001	0.08
No psychosis history	2.5 (1.6–3.9)	<0.001	

**Figure 2:**
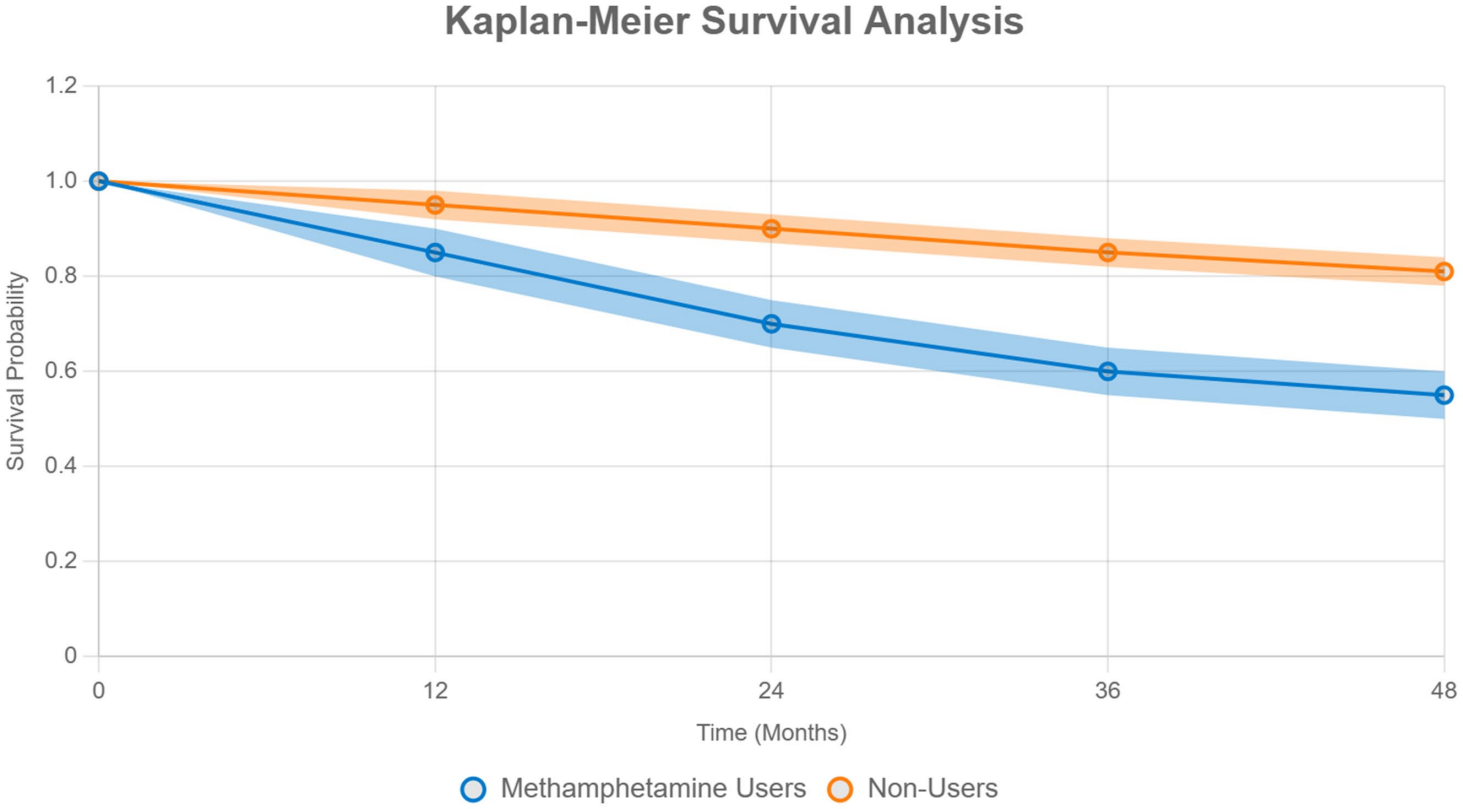
Kaplan–Meier survival analysis. Survival probabilities are depicted for both methamphetamine users (blue) and non-users (orange). Log-rank test: *χ*^2^ = 28.6, *p* < 0.001. Median time-to-event is 36 months for users and 48 months for non-users. Proportional-hazards assumptions were met (Schoenfeld residuals, *p* = 0.53). Shaded areas represent 95% confidence bands.

Kaplan–Meier survival analysis showed significantly lower survival probabilities for methamphetamine users compared to non-users (log-rank test, *χ*^2^ = 28.6, p < 0.001). Median time-to-event was 36 months for users and 48 months for non-users. Proportional-hazards assumptions were verified (Schoenfeld residuals, *p* = 0.53). Figure 2 displays survival curves with 95% confidence bands.

## 4 Discussion

This study identified a strong independent association between methamphetamine use and suicidality, which reflects the findings of previous studies that have linked SUDs to an increased risk of suicide (6, 11, 13). Methamphetamine users had considerably higher suicidality rates (30%) than those who used other substances (20%). These findings are consistent with those reported by Bayazit et al. (14), who found that suicidal thoughts were reported by more than 50% of methamphetamine-intoxicated patients seeking treatment in emergency settings. A lower rate of suicidality was observed in the present study; however, this disparity could be attributed to differences in the study populations, as the present population comprised patients from mental health facilities rather than emergency departments who may have been more intensely intoxicated. In contrast, data from the Saudi National Mental Health Survey showed that among the general population, the lifetime prevalence of suicidal ideation was just 4.90%, with only 1.46% of individuals attempting suicide, emphasizing that methamphetamine use elevates suicide risk compared to that in the general population (8). This distinction emphasizes the importance of suicide risk assessment and prevention among methamphetamine users.

Most of the participants in the study were male and had a mean age of 32.4 years; these findings align with those of Darke et al. (15), who found that 77% of methamphetamine-related suicides involved males, with an average age of 33.1 years. This consistency suggests that young men are more likely to use methamphetamine, possibly due to genetic and neurobiological factors (16), increased risk-taking behaviors, impulsivity, and societal stigma because drug use contradicts traditional gender norms associated with femininity. Furthermore, women who develop SUDs may encounter considerable barriers when seeking treatment, such as financial constraints, decreased readiness for abstinence, and family responsibilities (17, 18).

The methamphetamine users in this study exhibited a suicidal profile distinct from that of non-users, with significantly higher rates of suicide planning and suicide. This pattern is consistent with that reported by Darke et al. (15), who found that approximately 25% of methamphetamine-related suicides involved prior suicide attempts. Similarly, Zweben et al. (19) observed that 27% of methamphetamine users had made prior suicide attempts, indicating a strong relationship between impulsivity and suicidality. Chronic methamphetamine use has been linked to significant changes within dopaminergic and serotoninergic pathways of the brain, as well as neurotoxic effects within the striatum and the frontal lobe. Such changes may impair emotional regulation, executive function, and impulse control, while also worsening psychiatric comorbidities, leading to rapid transitions from distress to suicidal action (20–22). Müller et al. (23) also emphasized that cognitive dysfunction, increased aggression, and impaired decision-making further exacerbate suicide risk in methamphetamine users.

This study found a significant dose–response relationship between the frequency of methamphetamine use and suicidality. The survival analysis revealed that methamphetamine users had significantly lower survival rates compared with those of non-users, indicating that they were more than twice as likely to attempt suicide over a given time interval. This demonstrates the cumulative effect of continuous methamphetamine use on mental health deterioration and suicidality, emphasizing the importance of long-term psychiatric follow-up evaluations and interventions. Furthermore, relapse prevention measures and ongoing monitoring are critical, even after periods of abstinence, to reduce the persistent risk of suicide in this vulnerable population (24).

Self-poisoning is a major method of suicide among individuals with SUDs. Recent research has indicated that opioids are associated with the highest risk of fatal self-poisoning, followed by barbiturates and antidepressants, which is consistent with the present findings, demonstrating a strong relationship between methamphetamine, opioid, and antidepressant use and self-poisoning attempts. This emphasizes the importance of identifying substance-specific hazards in overdose situations, as some considerably increase lethality (25).

A considerable percentage (16.8%) of the methamphetamine users had a history of psychosis, which is consistent with the 12.3% prevalence among methamphetamine-related cases reported in a previous study (15). This substantial link between methamphetamine use and psychosis indicates that psychotic symptoms, whether transitory or chronic in nature, may play a role in increasing the risk of suicide, potentially by causing distress, affecting one’s perception of reality, and inducing paranoia or auditory command hallucinations. Other studies have reported a greater rate of substance-induced psychosis (28.6%) among methamphetamine users, further reinforcing the role of the drug in disrupting dopamine regulation and contributing to psychotic symptoms (26).

In the MENA region, drug overdose has been reported to be the most common method of suicide (27). In contrast, the present study found that hanging was the most common method of suicide among methamphetamine users, indicating a shift toward more violent methods. This disparity could be due to the neurobiological effects of methamphetamine, which increases aggression and impulsivity and encourages poor decision-making, raising the likelihood of high-lethality suicide attempts.

### 4.1 Limitations and future research directions

Although the present findings indicate that methamphetamine use is an independent risk factor for suicidality, the case–control approach limits the ability to draw firm inferences regarding causality. Despite accounting for confounding variables, residual confounders may still exist among unmeasured factors. Although the study involved several mental health hospitals, the results may not be fully representative of individuals outside of psychiatric settings or may not be generalizable to larger groups owing to disparities in healthcare systems and sociocultural circumstances. Future studies should employ longitudinal or experimental designs and include more varied populations to further elucidate these correlations and validate any causal links.

### 4.2 Conclusion

Methamphetamine users exhibited significantly greater suicidality and lower survival rates compared to those of other substance users, demonstrating the drug’s independent contribution to suicide risk. Heavy methamphetamine use is associated with increased suicidality, and a high prevalence of psychosis among users compounds this risk. This study highlights the serious public health issue of methamphetamine-related suicidality, emphasizing the urgent need for comprehensive interventions to effectively reduce suicide risk in this patient population.

## Data Availability

The original contributions presented in the study are included in the article/supplementary material, further inquiries can be directed to the corresponding author.
